# Infrared pupillometry, the Neurological Pupil index and unilateral pupillary dilation after traumatic brain injury: implications for treatment paradigms

**DOI:** 10.1186/2193-1801-3-548

**Published:** 2014-09-23

**Authors:** Jefferson William Chen, Kiana Vakil-Gilani, Kay Lyn Williamson, Sandy Cecil

**Affiliations:** Department of Neurological Surgery, Legacy Emanuel Medical Center, 2801 N. Gantenbein Ave, Portland, OR 97227 USA; Department of Neurological Surgery, University of California Irvine, 200 S. Manchester Ave, Suite 210, Orange, CA 92868 USA

**Keywords:** Infrared pupillometry, Neurological Pupil index, Traumatic third nerve palsy, Pupillometer, Oculomotor nerve palsy, Traumatic brain injury, Pupillary outcome

## Abstract

Pupillary dysfunction, a concerning finding in the neurologic examination of the patient with an acute traumatic brain injury often dictates the subsequent treatment paradigm. Patients were monitored closely with an infrared pupillometer, with NPi technology, for acute changes in pupillary function. NPi technology applies a scalar value to pupillary function. A retrospective chart review was performed of traumatic brain injury patients with acute unilateral pupillary dilation, admitted to Legacy Emanuel Medical Center’s NeuroTrauma Unit, Portland, OR, and followed as outpatients, between January 2012 and December 2013. Clinical exam findings of pupillary size, NPi scores, and brain Magnetic Resonance Imaging and Computed Tomography images were analyzed. Five traumatic brain injury patients were identified with unilateral pupillary dysfunction with long-term follow-up after the initial injury. Each patient was monitored closely in the trauma bay for neurological deterioration with a pupillometer and the clinical exam. Two patients underwent subsequent intracranial pressure monitoring based on a deteriorating clinical scenario, including consistent abnormal unilateral NPi scores. One patient with consistent abnormal NPi scores and an improved clinical exam did not undergo invasive interventions. Two patients showed early improvement in NPi scores correlating with the normalization of their pupillary reactivity. Anisocoria improved in all patients despite concurrent abnormal NPi scores. Magnetic Resonance Imaging and Computed Tomography imaging studies, with a focus on the third nerve, revealed focal abnormalities consistent with the clinical findings. A unilateral blown pupil and abnormal NPi score in a traumatic brain injury patient are not necessarily indicative of intracranial pressure issues, and must be correlated with the entire clinical scenario, to determine the etiology of the third nerve injury and direct potential therapeutic interventions. Early NPi score normalization suggests pupillary function may improve. We found that NPi scores, as a component of the clinical exam, provide a sensitive, noninvasive and quantitative means of following pupillary function acutely and chronically after a traumatic brain injury.

## Background

According to the Centers for Disease Control, 1.7 million people in the United States suffer a traumatic brain injury (TBI) each year, and of these about 275,000 are hospitalized and 52,000 expire (Traumatic Brain Injury in the United States 2010). Initial clinical assessments of the TBI patient in the trauma bay and neurocritical care unit are important in directing neurosurgical interventions and neuroprotective strategies. Accurate and rapid neurological assessment can help minimize the effects of secondary brain injury (Martinez-Ricarte et al. 2013; Du et al. 2005; Boev et al. 2005; Kuo et al. 2011; Lowenstein and Loewenfeld 1958; Heller et al. 1990). Particular components of the initial assessment, pupillary size and reactivity to light, may provide information about potential impending intracranial problems, including intracranial pressure (ICP) issues and act as an early warning system for neurological deterioration (Martinez-Ricarte et al. 2013; Chen et al. 2011). Pupillary assessments, however, are often inconsistent and hard to correlate with the clinical exam because of the subjective and variable nature of these assessments (Du et al. 2005; Boev et al. 2005; Kuo et al. 2011). Infrared pupillometry (IP) introduced by Lowenstein and Loewenfeld (Lowenstein and Loewenfeld 1958) in the late 1950s eliminated many of the discrepancies from the eye exam by consistently measuring accurate pupillary size changes. Current models of the pupillometer allow users to routinely track pupillary size changes over time and measure pupillary light reflex (PLR) variables (Heller et al. 1990; Larson and Muhiudeen 1995; Wachler and Krueger 1999; Volpe et al. 2000; Litvan et al. 2000; Fotiou et al. 2000; Manley and Larson 2002; Munoz Negrete and Rebolleda 2013).

The NeurOptics NPi-100 pupillometer, developed by NeurOptics, is a FDA approved handheld pupillometer which analyzes each variable of the PLR. This pupillometer can accurately grade a pupil’s response to light, under ambient lighting conditions, using an algorithm based on normalized variables of the PLR. These scores, known as the Neurological Pupil index (NPi) are set on a scale from 0 to 5, where pupillary scores falling above 3 are considered within the normal ranges (Boev et al. 2005; Chen et al. 2011). Pupillary function analyzed by IP in the neurocritical care setting eliminates intra- and interobserver variability and permits repeatable, noninvasive examinations of pupil size and reactivity (Martinez-Ricarte et al. 2013; Rouche et al. 2013; Ortube et al. 2013; Kau et al. 2007). Taylor and colleagues have shown that IP is a reliable and rapid way to assess pupillary function and asymmetry in patients suffering acute TBI (Taylor et al. 2003). With the additional quantitative pupillary function information, Chen and colleagues in a multicenter study, evaluated the efficacy of using NPi scores to follow patients with elevated intracranial pressure (ICP), finding a correlation between decreasing NPi scores and increased ICP (Chen et al. 2011). Their study, however, did not address situations of abnormal NPi scores in the absence of elevated ICPs.

Identification of the etiology of dilated and nonreactive pupils in acute TBI patients, without suspected ICP problems, relies on the clinical examination, in conjunction with Computed Tomography (CT) and Magnetic Resonance Imaging (MRI) studies of the brain (Brazis 2009; Sadagopan and Wasserman 2013; Lee et al. 2009; Murchison et al. 2011; Kwon et al. 2013). Imaging studies are particularly helpful in patients with compromised sensorium in the neurocritical care unit. Brain images of the cavernous sinus and subarachnoid spaces have captured some of the third nerve abnormalities in patients with traumatic third nerve palsy (Quisling et al. 2006; Kwon et al. 2013). Isolated unilateral third nerve palsies, with normal brain studies, have been reported in up to 15% of all minor TBI cases (Chen et al. 2005; Muthu and Pritty 2001; Najafi and Mehrbod 2012; Kwon et al. 2013). Najafi and Mehrbod (Najafi and Mehrbod 2012) describe a minor TBI patient with a third nerve palsy, who demonstrated improvement of third nerve function six months post injury. Non-contrasted cranial MRI and CT studies failed to demonstrate any abnormalities that explained the unilateral nonreactive dilated pupil. Similar case studies suggest traumatic third nerve palsy may occur through either direct mechanisms including distraction of the nerve, fascicular damage, and decrease in blood supply or indirect mechanisms such as nerve compression and displacement by a lesion (Chen et al. 2005; Muthu and Pritty 2001; Najafi and Mehrbod 2012). Oftentimes outcome of traumatic third nerve injury is guarded with limited full recovery, leaving patients with compromised vision, light sensitivity and physical deformities (Sadagopan and Wasserman 2013; Lin et al. 2013).

Several studies have focused on the correlation between initial pupillary function assessment (size and reactivity) and overall outcome prognosis in TBI patients (Kuo et al. 2011; Manley and Larson 2002; Lieberman et al. 2003; Chesnut et al. 1994; Marshall et al. 1983). Most of these studies were directed at patients with increased ICP issues. Our study is unique in quantitatively describing changes in pupillary sizes and NPi scores in patients presenting with unilateral pupillary dilation, with normal or abnormal ICPs, throughout their inpatient stays and follow-up appointments. The underlying etiology of the traumatic nerve palsy may be attributed to many considerations, including mechanical or non-mechanical damage to the nerve or more devastating circumstances like elevated ICP. Monitoring initial pupillary function is especially important for TBI patients suffering third nerve injuries, as this may affect the treatment paradigm for the brain injury and subsequent third nerve palsy (Lin et al. 2013). For example, the treatment of a unilateral pupillary dilation from nerve stretch is observation. In contrast, dilation of a pupil from a temporal epidural hematoma or elevated ICPs leads to invasive measures including craniotomy and hematoma removal. Additionally, because the recovery rate and outcome of a third nerve injury is variable, the NPi algorithm provides a sensitive and objective means to track changes in pupillary asymmetry and NPi scores over time (Chen et al. 2011; Boev et al. 2005; Lin et al. 2013).

The third cranial nerve controls movement of four of the six eye muscles involved with extraocular movements, pupillary constriction, position of the upper eyelid, and ability of the eye to focus; thus, chronic long-term nerve dysfunction can leave patients with ptosis, strabismus, and diplopia. A poorly constricting pupil can lead to photophobia. Moreover, physical appearance of this palsy may add psychological stress (Prasad and Volpe 2010; Brazis 2009). Surgical interventions for traumatic third nerve palsy post TBI, have improved some of the nerve palsy symptoms, however, success is limited (Lin et al. 2013). Better results may be achieved through early intervention and understanding of the specific underlying etiologies (Sadagopan and Wasserman 2013; Lin et al. 2013). Acute IP studies on TBI patients with unilateral third nerve palsy may help identify specific markers of third nerve function. Furthermore, the long term follow-up and imaging studies, in particular MRI studies, may provide information about the mechanism(s) of the third nerve injury and prognosis for pupillary recovery.

## Results

### Baseline characteristics

Characteristics of the five traumatic brain injury (TBI) patients with unilateral third nerve injuries (three female, average age of 43.2 ± 21.5 years, range, 12 to 67 years) are summarized in Table 1. All patients were placed on opioids and benzodiazepines (except patient two) during the initial intensive care unit observation. Patient three was also placed on a barbiturate during the early part of his evaluation. Patient two was on anticoagulant therapy at the time of the injury. Average admission Glasgow Coma Scores (GCS) were 7.8 ± 5.7 (range, 3 to 15) and Glasgow Outcome Scores (GOS) were 5 across the group. Patients three and five underwent invasive intracranial pressure (ICP) monitoring for suspected ICP problems. Patient three also underwent evacuation of the traumatic basal ganglia hemorrhage on the side opposite the dilated pupil because of the local mass effect from the blood clot. His ICP was in the 20 mmHg range pre-operatively. Each of the patients had serial pupillometry measurements during hospitalization and received further evaluations at each consecutive follow-up visit. Length of long-term follow-up varied with each patient with an average of 7.6 ± 6.8 months (range, 2 to 15 months).

**Table 1:** **Baseline characteristics and CT/MRI findings of five patients with traumatic brain injury and unilateral third nerve injury**

Patient #	Trauma mechanism	Age/sex	GCS	Initial CT trauma findings relative to the injured third nerve	CT/MRI abnormalities in the region of the affected third nerve	Surgical intervention	Follow- up time (months)	Pupillary function outcome
1	Bicycle versus automobile	43/F	5	Contralateral frontal contusion	MRI: enhancement of cisternal portion	None	15	Normal: R pupil function returns slowly
2	Ground level fall	67/M	15	Perimesencephalic bleed	CT: blood adjacent to the third nerve	None	2	Normal: R pupil function returns rapidly
3	Bicycle accident	12/M	3	Contralateral BG hemorrhage	MRI: enhancement of cisternal portion	Left sided evacuation of BG bleed	15	Abnormal: R pupil dilated and unresponsive
4	Thrown from horse	35/F	13	None	MRI: enhancement of cisternal portion	None	4	Abnormal: L pupil anisocoria, dilated, and unresponsive
5	Motorcycle accident	59/F	3	R CCF, orbital rims fractured	CT: CCF	Endovascular obliteration of CCF	2	Abnormal: R pupil dilated and unresponsive

Each patient recovered normally and was assessed as a Glasgow Outcome Score of 5.

BG basal ganglia, CCF carotid cavernous fistula, CT computed tomography, GCS Glasgow Coma Score, L left, MRI magnetic resonance imaging, R right.

### Infrared pupillometry findings

**Figure 1:**
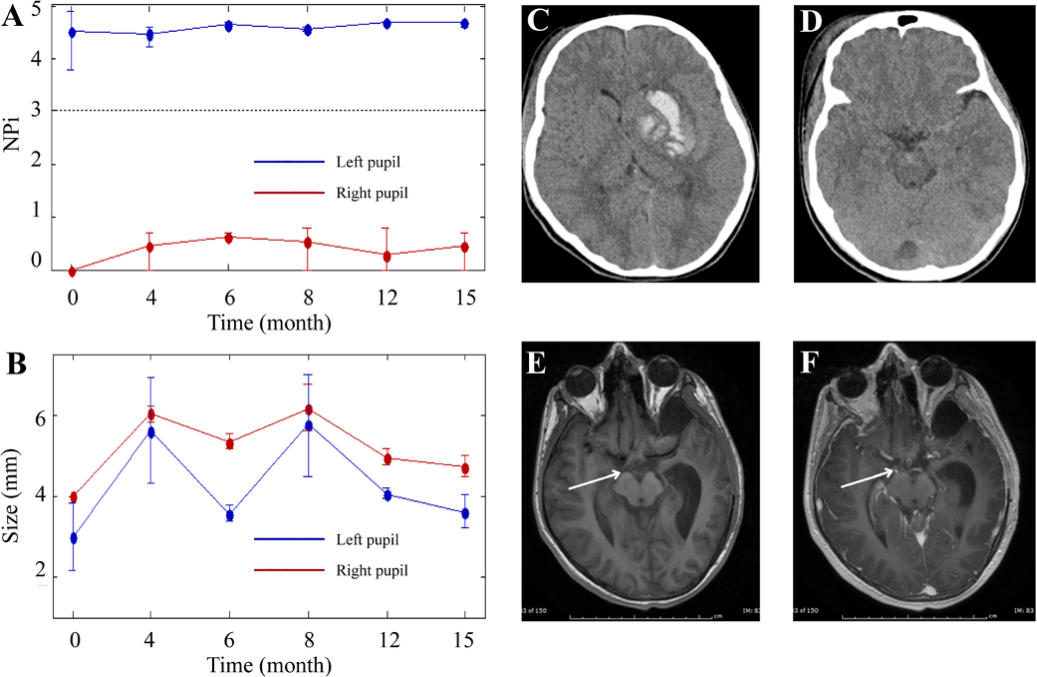
**No return of pupillary function: relationship of NPi scores, pupillary asymmetry, and brain images.** Patient three is a 12-year-old helmeted male motorcross cyclist involved in a crash. Initial CT studies **(C, D)** revealed a left-sided basal ganglia hemorrhage. Initial IP NPi scores (**A**, time 0 months) and IP size measurements (**B**, time 0 months) show some asymmetry between the pupils and no pupillary function on the right side. IP assessments from follow-up appointments (**A**, **B**, time 4-15 months) indicate some return of pupillary symmetry between both pupils and nominal increases in NPi values for the right pupil by month four. Post-contrast MRI studies at six weeks **(F)** capture enhancement of the third nerve (see white arrow) at the location where the nerve leaves the midbrain. Non-contrasted MRI studies **(E)** show the right intact nerve at six weeks. Note: the time = 0 months represents the average of hourly measurements that were done during the first week that the patient was in the ICU. The measurements taken at the other time points were done in the NeuroTrauma clinic and represent an average of three measurements.

**Figure 2:**
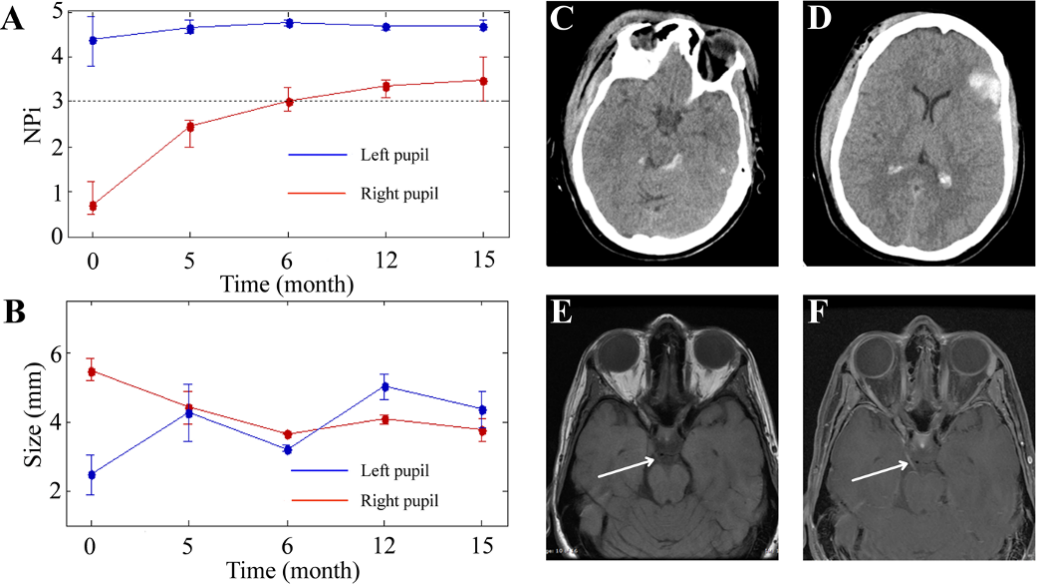
**Partial return of pupillary function: relationship of NPi scores, pupillary asymmetry, and brain images.** Patient one is a 43-year-old unhelmeted female bicyclist involved in a collision with a stationary automobile. Initial CT studies **(C, D)** revealed a left-frontal intracerebral contusion. Initial IP NPi scores (**A**, time 0 months) and IP size measurements (**B**, time 0 months) show significant third nerve dysfunction on the right side and asymmetry between the pupils. IP assessments from follow-up appointments (**A**, **B**, time 5-15 months) indicate a return of pupillary symmetry between both pupils and slow, incremental increases in NPi scores for the right pupil. Non-contrasted MRI studies at three months show the right intact third nerve (**E**, see white arrow) and post-contrast MRI studies at three months **(F)** demonstrate enhancement of the third nerve (see white arrow) at the location where the nerve leaves the midbrain. Note: the time = 0 months represents the average of hourly measurements that were done during the first week that the patient was in the NeuroTrauma ICU. The measurements taken at the other time points were done in the NeuroTrauma clinic and represent an average of three measurements.

**Figure 3:**
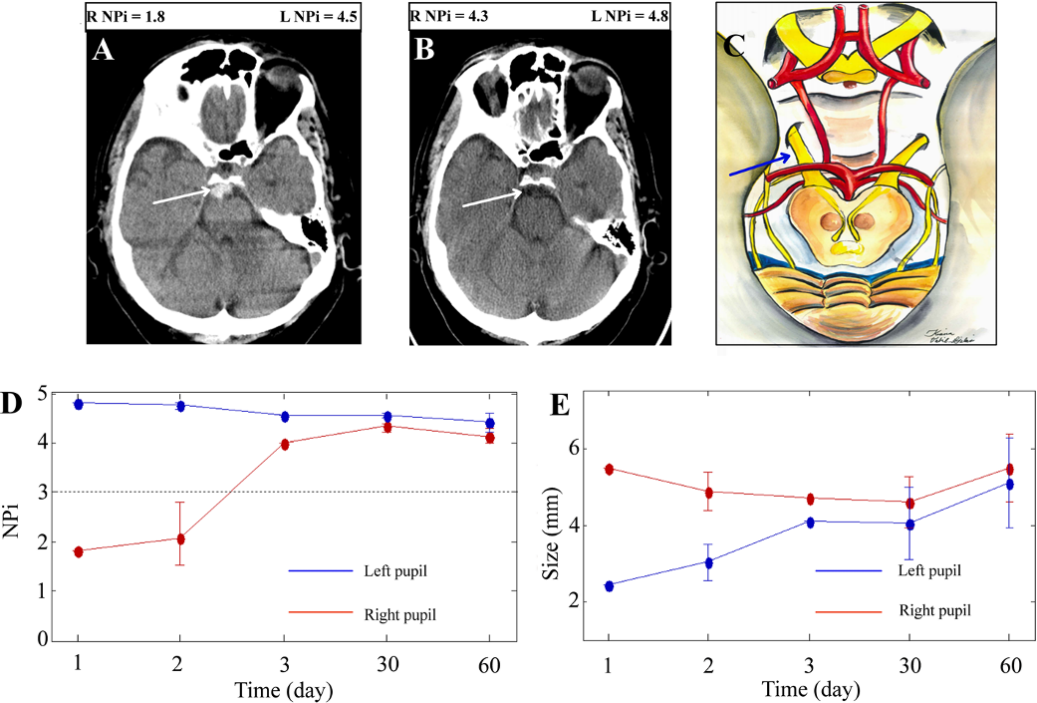
**Complete return of pupillary function: relationship of NPi scores, pupillary asymmetry, and brain images.** Patient two is a 67-year-old male taking Coumadin who suffered a ground level fall. Initial CT studies **(A)** revealed a hemorrhage in the prepontine cistern (white arrow denotes blood) and one month follow-up CT studies **(B)** showed dissipation of the hemorrhage and a visible third nerve (white arrow). Initial IP NPi scores (**D**, time 1 day) and IP size measurements (**E**, time 1 day) show third nerve dysfunction on the right side and asymmetry between the pupils. By day three NPi scores **(D)** have significantly increased and the pupils are symmetric **(E)**. The top, right schematic **(C)** depicts the anatomy of the third cranial nerves as they leave the midbrain and enter the cisternal space enroute to the cavernous sinus. Blue arrow **(C)** indicates the presumed region of third nerve compression by the blood clot. Note: the time = 0 days represents the average of hourly measurements that were done during the first few hours that the patient was in the ICU. The measurements taken at the other time points were done in the NeuroTrauma clinic and represent an average of three measurements.

All pupillometer measurements were taken under similar ambient lighting conditions in the intensive care unit and outpatient clinic. Baseline admission NPi scores from patients three through five (see Figure 1A, patient three) were recorded as zero for the affected pupil. Subsequent measurements taken during the last follow-up appointment on these patients demonstrated minimal change (NPi = 0.4 at 15 mo, 0.2 at 4 mo, and 0.0 at 2 mo, respectively), while their ophthalmologic abnormalities (ptosis and diploplia) improved and the pupil sizes converged symmetrically. Patients’ one (Figure 2) and two (Figure 3) demonstrated a return of pupillary function over two different time courses: gradual and rapid, respectively. Patient two had an initial NPi score of 1.8 and by day three pupillary functions had returned to normal, measured with an NPi score of 4.0. At two months, pupil function remained clinically normal, (NPi = 4.1), pupil sizes had converged, and all other ophthalmologic abnormalities had resolved. Patient one had an initial NPi score of 0.7 and follow-up measurements over the course of fifteen months showed slow improvements (NPi = 2.5 at 5 mo, NPi = 3.0 at 6 mo, NPi = 3.4 at 12 mo and NPi = 3.5 at 15 mo) and some photophobia in the affected eye. All five patients demonstrated functional improvement of their oculomotor muscles and ptosis over the course of long-term follow-up visits.

### Diagnostic imaging findings

Computed Tomography (CT) and Magnetic Resonance Imaging (MRI) studies suggested abnormalities in the region of the affected third nerve in all five patients. In patients one (Figure 2F), three (Figure 1F), and four, enhancement of the third nerve was noted on the post-contrast MRI images on the same side as the pupillary dysfunction at three months, six weeks, and four months, respectively. No abnormalities were noted on the images in the absence of the gadolinium. The caliber of the third nerves did not appear to be abnormal. Enhancement of the affected third nerve was not seen in follow-up MRI studies taken at six and twelve months (images not shown). Damage to the third nerve in these patients was likely caused by stretching of the nerve during the initial trauma. Patient two had a pacemaker in place and was evaluated only with CT. The hemorrhage seen in the region of the right third nerve in the prepontine cistern (Figure 3A) was likely to have exerted local mass effect on the nerve leading to the observed dysfunction. This blood clot dissipated and was not seen on the two month follow-up CT image (Figure 3B). Patient four had no indication of brain trauma on her initial CT and MRI studies images to indicate the cause of her acute third nerve injury. Her four month follow-up MRI studies, with contrast, demonstrated enhancement of the third cranial nerve in its cisternal course (not shown), similar to the patterns seen with patients one and three. Patient five had CT studies, CT angiograms, and cerebral angiograms that demonstrated a large cavernous-carotid fistula. Several embolization procedures decreased the aberrant arterio-venous flow. The patient had improvement in the ptosis, proptosis, and extra-ocular movements, but no improvement in pupillary function after the embolizations.

## Discussion

Pupillary size, symmetry, and reactivity to light are important components of the neurological assessment of traumatic brain injury (TBI) patients. Abnormal pupils (asymmetry and non-reactivity) are common, but, important dilemmas in the triage of these patients. Some of the more frequent considerations in the differential diagnosis of a unilateral dilated pupil include elevated intracranial pressure (ICP), direct third nerve stretch from the energy of the injury, a brain stem contusion in the region of the third nerve nuclei, a blood clot in the cisterns surrounding the third nerve, an aneurysm of the posterior communicating artery, a mass lesion in the adjacent temporal lobe, and a cavernous carotid fistula (Chen et al. 2005; Muthu and Pritty 2001; Najafi and Mehrbod 2012; Ritter et al. 1999; Sadagopan and Wasserman 2013; Motoyama et al. 2012; Guresir et al. 2012; Prasad and Volpe 2010; Kim et al. 2009; Yanovitch and Buckley 2007; Nistri et al. 2007; Bhatti et al. 2006; Chen et al. 2011). Indeed, the etiology of the dilated or nonreactive pupil will dictate evaluation and treatment paradigms (Lin et al. 2013). In this retrospective review study, we present infrared pupillometer (IP) documentation on some of the causes of pupillary asymmetry and non-reactivity that were mentioned above. We also describe the utility of using IP with Neurological Pupil index (NPi) technology in the treatment paradigm of TBI patients with unilateral third nerve palsy.

Manual pupillary assessment protocols use a penlight to evaluate pupil reactivity and a ruler to measure pupil size, while IP removes this intra- and interobserver variability and permits repeatable, noninvasive examinations. Asymmetric pupils measuring greater than 1 mm in difference are considered pathologically significant. Reliance on ruler measurements of pupillary size for symmetry comparisons, is subjective and entirely dependent on the skill of the observer (Munoz Negrete and Rebolleda 2013). Subjectivity of size measurements leads to inconsistencies in the medical record of the patient’s symmetry measurements (Martinez-Ricarte et al. 2013; Du et al. 2005; Boev et al. 2005; Taylor et al. 2003; Rosenberg et al. 2011; Fountas et al. 2006; Hults et al. 2006). Pupillary measurements taken by the IP decrease discrepancies between clinicians (Boev et al. 2005) and add objectivity to the examination in the neurocritical care setting (Hallman and Joffe 2013). Our data show that IP readily captures pupil size (Figures 1B, 2B, and 3E) in mm, however, NPi technology is a more reliable method for evaluating pupillary function. Pupillary size measurements assess pupillary symmetry/asymmetry, however, other components of the pupillary light reflex (PLR) are not evaluated (Taylor et al. 2003; Chen et al. 2011). Pupils are often categorized and recorded in the patients’ history as “sluggish” or “nonreactive,” however, this is an incomplete evaluation of pupillary function in contrast to the PLR variables included in the NPi algorithm.

Each patient in our study was evaluated upon presentation in the trauma bay by the resuscitation team using the standard penlight and ruler methodology. Electronic medical records (EMR) show that these patients were initially identified with a unilateral nonreactive and dilated pupil, however, immediate reassessment with IP in the intensive care unit (ICU) indicated patients’ one and two retained some conserved function in the affected pupil. This was demonstrated by initial NPi scores above 0.5 (Figures 2A and 3D). PLR has several different components (Taylor et al. 2003; Chen et al. 2011), which are summated by the NPi as a scalar value for pupillary function. NPi technology allows the quantitative assessment of pupillary asymmetry and pupillary reactivity in these TBI patients. Concerns for drug interactions with pupillometer recordings were raised, however, because of the unilateral pupillary dysfunction, other etiologies were considered for the third nerve palsy, as drug effects (particularly those from barbiturates) would likely affect both pupils (Larson et al. 1997; Rollins et al. 2014; Belani et al. 1993; Hou et al. 2007).

**Figure 4:**
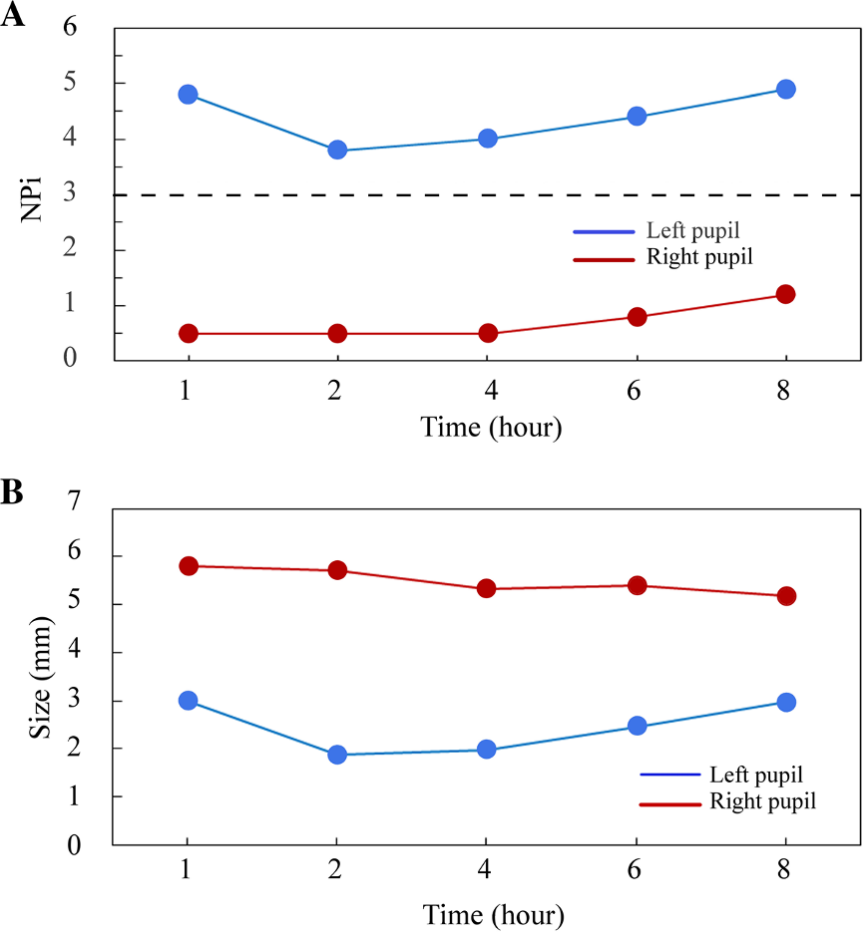
**Relationship of NPi scores and pupillary asymmetry in the acute treatment course.** Patient one’s initial eight hour pupillary function time-course, post traumatic brain injury, in the NeuroTrauma intensive care unit. Her right NPi scores **(A)** gradually improved from 0.5 to 1.2, while her left NPi scores stayed within normal ranges. Her pupillary asymmetries **(B)** over the same time frame remained significantly different. Initial improvement in her right NPi scores guided an observational treatment paradigm over neurosurgical invasive therapies.

Patient one is a good example of how NPi scores altered the course of the patient’s care. She presented with a Glasgow Coma Score (GCS) of 5 and was intubated and sedated. She also had a right dilated pupil assessed by a ruler and pen light. Her initial Computed Tomography (CT) study revealed a left frontal traumatic contusion (Figure 2D) and areas of diffuse axonal injury with some blood in the left perimesencephalic cistern, however, these cisterns were open and non-compressed. Her initial abnormal NPi score for the right pupil and the neurological exam raised questions about impending ICP issues, however, repeated NPi measurements showed improvement in her pupillary function (Figure 4A) over the course of eight hours post TBI. Her pupillary function recorded by the pupillometer was overlooked in the initial manual exam assessments. Over the same acute monitoring period her pupillary asymmetry did not improve (Figure 4B). ICP monitoring and surgical evacuations were considered, however, because of the improvement in her right NPi scores, sedation was decreased and she awoke, followed commands and was extubated according to protocol.

Patients three through five with initial NPi scores of 0 (Figure 1A) saw minimal improvement in pupillary reactivity at long-term follow-up visits (see Table 1). Conversely, patients one and two had small initial NPi numbers (NPi = 0.7, [Figure 2A] and 1.8, [Figure 3D] respectively), and saw significant improvement of their third nerve function over two distinct improvement timelines: rapid and gradual. Patient two had a quick third nerve recovery, with baseline NPi = 1.8, regaining full nerve function by day three post TBI (Figure 3D). Patient one demonstrated a partial recovery, with an initial NPi = 0.7, gradual improvement of NPi scores to 1.2 over eight hours post TBI, and her pupillary function was within normal limits (Taylor et al. 2003; Chen et al. 2011) (Figure 2A) at her fifteen month follow-up visit. Although our sample size is small, these preliminary NPi scores and functional pupil recovery correlations suggest NPi is a valuable clinical tool for following traumatic third nerve injuries. These correlations further suggest early improvement in NPi scores may be associated with shorter recovery periods and better functional outcome for pupillary function.

Each patient also received MRI/CT imaging studies with special attention to the third nerve. Previous research demonstrates that patients suffering a mild to severe TBI, involving pupillary nerve fiber dysfunction, may display a range of functional return of the third nerve at long-term follow-up visits (Najafi and Mehrbod 2012; Sadagopan and Wasserman 2013; Brazis 2009; Lin et al. 2013). Furthermore, radiology reports have indicated that nerve injuries are not always visualized on brain images, although newer imaging techniques can sometimes capture the lesions in close proximity to the third cranial nerve (Kau et al. 2007; Quisling et al. 2006; Sadagopan and Wasserman 2013; Pula et al. 2011; Chaudhary et al. 2009; Lee et al. 2009; Saremi et al. 2005; Garcia-Rivera et al. 2001; Kwon et al. 2013). Our data highlights that in some cases third nerve injury due to trauma is visible only on the post-contrast MRI images. As seen in Figures 1F and 2F, enhancement is visible along the third nerve cisternal segment. This nerve enhancement was not seen at their six and twelve month follow-up study (not shown). Of note, enhancement of the cisternal portions of the third nerve seen in patients one, three, and four was time limited and consistent with an inflammatory process (Quisling et al. 2006; Pula et al. 2011; Lee et al. 2009; Saremi et al. 2005; Garcia-Rivera et al. 2001). Patient four had an early MRI study within 48 hours of the injury that did not demonstrate any enhancement, however, enhancement was seen on her four month follow-up study, suggesting development of an inflammatory process over time.

Patient one’s pupillometry studies demonstrated that the pupil asymmetry was minimal (Figure 2B) and the improvement in NPi scores had plateaued (Figure 2A). It is interesting that her injury (Figure 2C,D) shows a frontal contusion on the left, which is contralateral to the pupillary abnormality. Similarly, in patient three, the site of brain injury is contralateral to the pupillary dysfunction. These findings suggest that the third nerve injuries in these cases were related to the stretch or torque of the third nerve as opposed to direct pressure from an expanding hemorrhage in the adjacent temporal lobe.

The observations reported here on utilizing the NPi scale in neurocritical care settings demonstrate the clinical applications for pupillary index technology. Consistent with previous reports (Chen et al. 2011; Boev et al. 2005; Taylor et al. 2003), the pupillometer provides a quantitative method for determining pupillary symmetry and function, and for following associated changes over time. Our results demonstrate that the NPi scale is a more sensitive measure of pupillary function in TBI patients over pupillary asymmetry, recorded either manually or with IP. These findings also suggest that early improvements in NPi scores correlate with a better prognosis for the return of pupillary function and associated third nerve abnormalities. This is particularly important when prognosticating to the patient early in their neurocritical care course. Early improvements in NPi scores may project more hope for recovery and thus warrant early aggressive neuro-ophthalmology therapy aimed at reducing chronic ocular dysfunctions secondary to the initial trauma (Sadagopan and Wasserman 2013; Lin et al. 2013). The findings of MRI third nerve enhancement patterns, consistent with inflammation and the subsequent resolution of the enhancement, suggests the diminution of the inflammatory process. This may represent the endpoint of the inflammatory effects on third nerve function.

## Conclusion

Pupillary function index technology, used in conjunction with the clinical exam, offers a robust measure of pupillary function in the neurologically impaired patient. A unilateral non-reactive pupil, after traumatic brain injury, is not necessarily indicative of increased intracranial pressure or drug interactions. Careful examination of these patients is crucial for identifying the underlying etiology of the traumatic third nerve palsy, and in turn directing clinical management. Initial pupillometer measurements in the intensive care unit are useful in predicting whether or not pupillary function will improve. The data from our patients demonstrates that early improvement of NPi scores is a favorable indicator for the return of pupillary function, regardless of the presence of symmetric/asymmetric pupils.

## Methods

### Inclusion/exclusion criteria

This was a retrospective chart review of patients diagnosed with a traumatic brain injury (TBI) and unilateral pupillary dysfunction admitted to the NeuroTrauma services at Legacy Emanuel Medical Center (LEMC), Portland, OR, and followed in the associated outpatient NeuroTrauma clinics from January 2012 through December 2013. As this was a retrospective chart review, LEMC’s Institutional Review Board provided expedited approval of the study and waiver of consent. ICD-9 codes and keywords that were searched included traumatic brain injury, unilateral dilated pupil, complete/incomplete unilateral third nerve palsy, and NPi. Patients were identified with TBI, anisocoria and a unilateral third nerve palsy that included abnormal pupillary function and either aberrant extraocular movements, ptosis, or both. Pupillary function and size were measured using the NeurOptics NPi-100 Pupillometer. This pupillometer utilizes the Neurological Pupil index (NPi) algorithm, which is based on normative data and grades the pupil’s response between 0 and 5; values ranging between 3 and 5 correspond to the normal variation observed in the healthy population. In normal conditions the left and right NPi scores of both pupils should be above 3 and similar, and the pupils should be symmetrical. Pupillometer measurements were taken under similar ambient lighting conditions during the patients’ impatient stays and outpatient follow-ups. Of note the pupillometer reliably has been demonstrated under diverse lighting conditions and accurately used on patients under sedation and ventilated (Boev et al. 2005; Rouche et al. 2013; Martinez-Ricarte et al. 2013; Behrends et al. 2012; Fountas et al. 2006; Hults et al. 2006; Meeker et al. 2005; The Brain Trauma Foundation. The American Association of Neurological Surgeons. The Joint Section on Neurotrauma and Critical Care. Pupillary diameter and light reflex 2000).

### Protocol

All patients were initially assessed at admission by a board certified neurosurgeon. Computed Tomography (CT) and/or Magnetic Resonance Imaging (MRI) studies were obtained as part of the neurological workup determining brain injury severity and etiology of the pupillary abnormality. Patient five had additional CT angiogram and conventional angiogram studies during the embolization procedures to obliterate her carotid cavernous fistula. Pupillary function and size measurements for each patient were routinely examined by the intensive care unit nurses and taken during outpatient visits. Assessments made with both the manual penlight and the infrared pupillometer were recorded in the Electronic Health Records (EHR) system as part of the patient’s routine medical care. Patient data including demographic information, medical history, pre-treatment Glasgow Coma Scores, discharge Glasgow Outcome Scores, operative reports and MRI/CT studies were gathered from the EHR.

### Data analysis

Pupillometer data, including NPi scores and pupillary size, were analyzed using MATLAB software. Standard deviation bars in Figures 1, 2 and 3 reflect the average of multiple IP measurements of NPi and pupil size taken during initial assessment and follow-up. CT and MRI findings were correlated with the time points of the pupillometer recordings in relationship to the clinical course.

## Abbreviations

EHR: Electronic Health Records
GCS: Glasgow Coma Score
GOS: Glasgow Outcome Score
ICU: Intensive Care Unit
IP: Infrared pupillometry
NPi: Neurological Pupil Index
PLR: Pupillary light reflex
TBI: Traumatic brain injury.
